# Establishing a Dementia Prevention Framework in Rural India: The SMRUTHI Cohort's Baseline View

**DOI:** 10.1002/alz70861_109026

**Published:** 2025-12-23

**Authors:** Hina Narzari, Anu Gupta, Nilima N, Shubham Gupta, Kritika Sharma, Aditi Dubey, Varuna Sharma, Sakshi Sharma, Kajal Fulara, Sneha, Kaamini Kashyap, Shikha Chaudhary, Krishan Kaushal, Phaniraj Vastrad, Priya darshanraj N, Sandip Bhattacharjee, Gajraj Singh Shekhawat, Harshath Ajay V, Sandesh J R, Ambika YV, Bishakha Sen, Shalina Jamatia, Priyanka Kumari Meena, Hitesh Tiwari, Shaily Bhushan, Sagar PM, Ripanjit Singh, Manish Acharjee, Kalpna Sharma, Aishwarya Bhovi, Mailarappa Padiyappa Hooli, Shilpa K, Naveen MR, Dipankar Deb, Pameli Jamatia, Pooja Sharma, Akhilesh Nagar, Tinku Yogi, Rishav Bhatia, Rishabh Gautam, MA Khan, Vaibhav Patil, Rajeev Aggarwal, Ashima Nehra, Subarna Roy, Manish Barvaliya, Subrata Baidya, Shampa Das, P K Anand, Sunil K Raina, Abhik Sinha, Venugopalan Y Vishnu, MV Padma Srivastava

**Affiliations:** ^1^ All India Institute of Medical Sciences, New Delhi, Delhi India; ^2^ All India Institute of Medical Sciences, Delhi, Delhi India; ^3^ All India Institute of Medical Sciences, New Delhi India; ^4^ All India Institute of Medical Sciences, New delhi, Delhi India; ^5^ MRHRU, Raichur, Karnataka India; ^6^ MRHRU, Khumulwmg, Tripura India; ^7^ Model Rural Health Research Unit (MRHRU), Jaipur, Rajasthan India; ^8^ Model Rural Health Research Unit (MRHRU), Sirwar, Raichur India; ^9^ Model Rural Health Research Unit (MRHRU), Sirwar, Raichur, Karnataka, India, Karnataka India; ^10^ Model Rural Health Research Unit (MRHRU), Khumulwng, Tripura India; ^11^ MRHRU, Jaipur, Rajasthan India; ^12^ Department of Community Medicine, Dr. RP Government Medical College, Tanda, Himachal Pradesh, India, Tanda, Himachal Pradesh India; ^13^ MRHRU, Sirwar, Karnataka India; ^14^ f) Department of Community Medicine, Dr. RP Government Medical College, Tanda, Himachal Pradesh, India, Una, Himachal Pradesh India; ^15^ Model Rural Health Research Unit (MRHRU), Raichur, Karnataka India; ^16^ MRHRU, Una, Himachal Pradesh India; ^17^ ICMR‐NITM, Belagavi, MRHRU Sirwar, Raichur, Karnataka India; ^18^ In charge MRHRU Khumulwmg, Tripura India; ^19^ Community Medicine AGMC, Khumulwng, Tripura India; ^20^ ICMR‐NIIRNCD, Jodhpur & Nodal Officer, MRHRU Bhanpur Kalan, Jaipur India; ^21^ Department of Community Medicine, Dr. RP Government Medical College, Tanda, Himachal Pradesh India; ^22^ ICMR Center for Ageing and Mental Health (CAMH), Kolkata India

## Abstract

**Background:**

With the projected surge in dementia cases across low‐ and middle‐income countries, India faces an urgent need for early prevention strategies targeting at‐risk populations. The SMRUTHI cohort was developed to characterize dementia risk factors and cognitive health among rural elderly individuals (aged ≥55 years) across diverse Indian regions.

**Method:**

This multicentric, cross‐sectional study recruited 2,402 participants across four rural regions: Tripura (East), Rajasthan (West), Karnataka (South), and Himachal Pradesh (North). Trained field investigators and psychologists conducted home visits and collected data using validated Case Report Forms (CRFs) via the REDCap platform. A dedicated data management team performed daily checks to ensure completeness and consistency. Participants were classified as illiterate if they were unable to read and write a short, simple statement related to daily life. Missing data were minimized through mandatory REDCap fields, planned revisits, and reassurances about confidentiality. Multiple imputation by chained equations (MICE) was planned for any remaining missing data. Monthly backups were done by the central statistician to ensure data security. Blinding was maintained; outcome assessors and the statistical analyst remained unaware of group allocation during analysis.

**Result:**

Among the 2,402 participants, most were aged ≥60 years (75.4–81.5%), and females comprised 58–66% across sites. Illiteracy was highest in Tripura (81.8%) and Karnataka (77.8%). Hypertension and diabetes were observed in up to 40.2% and 16.6%, respectively. Smoking (41.1%) and alcohol use (41.3%) were highest in Tripura. High adherence to the MIND diet was found in 97.3% in Tripura, but only 17.9% in Rajasthan. Low physical activity (<600 MET‐min/week) was most common in Karnataka (58.6%). PHQ‐9 scores showed depressive symptoms ≥5 in up to 9.7% (Karnataka); non‐zero medians ranged from 1 (Himachal) to 6 (Tripura). Median ACE‐III scores varied by region: Tripura 78 (95% CI: 77–80), Rajasthan 84 (83–85), Karnataka 88 (88–89), and Himachal 87 (87–88).

**Conclusion:**

The SMRUTHI cohort provides critical baseline data on dementia risk in rural India, highlighting the need for region‐specific, multidomain prevention strategies in low‐resource settings.

